# Is Countermovement Jump Height and One Repetition Maximum Back Squat Associated With the Peak Force of a Front Kick With and Without Carried Load?

**DOI:** 10.1519/JSC.0000000000005128

**Published:** 2025-05-16

**Authors:** Vladan Oláh, Vít Třebický, Jan Maleček, Vladimír Michalička, Jacek Wąsik, Michal Vágner

**Affiliations:** 1 Department of Military Physical Education, Faculty of Physical Education and Sport, Charles University in Prague, Prague, Czech Republic;; 2 Department of Gymnastics and Combat Sports, Faculty of Physical Education and Sport, Charles University in Prague, Prague, Czech Republic;; 3 Department of Physiotherapy, Faculty of Physical Education and Sport, Charles University in Prague, Prague, Czech Republic;; 4 Institute of Physical Culture Science, Jan Dlugosz University of Czestochowa, Poland; and; 5 Department of Sports Games, Faculty of Physical Education and Sport, Charles University in Prague, Prague, Czech Republic.

**Keywords:** performance, strength, training, hand to hand combat, self-defense, martial arts

## Abstract

Oláh, V, Třebický, V, Maleček, J, Michalička, V, Wąsik, J, and Vágner, M. Is countermovement jump height and one repetition maximum back squat associated with the peak force of a front kick with and without carried load? *J Strength Cond Res* 39(8): 880–889, 2025—The front kick is a fundamental technique used in self-defense and hand to hand combat. This study aimed to test whether front kick performance, both with and without a 30-kg carried load, can be effectively estimated or tracked through commonly available testing procedures such as the countermovement jumps (CMJ) and one repetition maximum back squat (1RM BS). In 2 testing sessions, 21 male participants (mean age = 22.7 years, *SD* = 1.9) performed 1RM BS, 5 front kicks peak force (FK) without and with 30-kg carried load (FK30), and 3 CMJ without and with 30-kg carried load (CMJ30). A force plate measured the peak force (*N*) of the FK and FK30 and the height (cm) of the CMJ and CMJ30. Statistically significant correlations were found between CMJ and FK (*r* = 0.55 [0.225–1], *p* = 0.005) and CMJ and 1RM BS (*r* = 0.54 [0.219–1, *p* = 0.005]). Carried load reduced both CMJ height by 61% (*d* = 3.35 [2.543–∞], *p* < 0.001) and FK peak force by 23% (*d* = 1.33 [0.828–∞], *p* < 0.001), compared without load condition. A regression model using 1RM BS and CMJ explained 31.5% of FK variance, with only the height of CMJ being statistically significantly associated, *β* = 0.626, *p* = 0.015. The study demonstrates that although there is a relationship between explosive power and maximum strengths, FK is more related to CMJ height than 1RM BS. From a practical standpoint, the study suggests that explosive power shows a better association with the FK than the maximal strength. Thus, training programs focused on lower limb explosive power could allow athletes and military personnel to achieve higher FK. Combat sports trainers and hand-to-hand combat instructors may thus consider accordingly updating their training schemes, emphasizing plyometrics and other explosive training methods for enhancing the peak force of kicks.

## Abstract 2

Supplemental Digital Content is Available in the Text.

## Introduction

The front kick is a fundamental technique taught across martial arts or hand-to-hand combat systems in military personnel (13,16). In terms of execution, the front kick is a relatively simple yet challenging (40) ballistic movement (1,5,29). When mastered, it becomes a very potent technique (10). The performance in kicks is typically quantified using kinetic analyses. Measures such as peak force (2,47) and impulse (20) offer insights into a participant's efficiency during the impact phase (34), such as a potential for causing soft tissue damage (12) or exerting concussive forces (49). The peak force generated by a kick is pivotal for success in combat, with observed forces spanning from approximately 3–7 kN (37,59). Peak performance in any kick is influenced by various factors, such as anthropometric dimensions, strength, or the combatant's technique level and training (58).

In military settings (39,62), the front kick is used not only to incapacitate or repel an adversary (16) but is also used when the use of weaponry is not feasible or appropriate (57). Moreover, it can be used for utilitarian objectives, such as forcing doors to open or removing obstacles in emergent situations (61,65). In contrast to athletes, the required equipment of military personnel (e.g., backpacks, ballistic armor, ammunition) creates greater difficulty when executing movements such as the front kick, as the additional mass impairs maximum power, velocity, mobility, stability, and overall kick technique (11,60,62).

Previous studies showed that maximal strength training, explosive strength training, or a combination of both can improve kicking ability measures (7,50,55). Therefore, it is deemed a comprehensive training program for improving front kick performance should incorporate training for increasing maximal and explosive lower limb strengths (56,53). Evaluating training progress requires regular, accurate, and reliable monitoring and testing (27). Vertically mounted force plates are typically used to assess kick performance measures like peak force (41). However, access to these devices or resources for their acquisition may be limited for some researchers, trainers, and trainees. Maximal lower limb strength assessments are often conducted via one repetition maximum back squat (1RM BS) (15). Similarly, the countermovement jump (CMJ) testing is one of the most valid and frequent protocols for assessing lower limb explosive strength (33). The advantage of these 2 tests lies in their reliability and ease of implementation.

Our study has 2 interrelated aims. First, we want to investigate whether lower limb performance in 1RM BS and CMJ is associated with the front kick peak force (FK) and whether 1RM BS and CMJ can serve as potential proxies for gauging FK performance. Second, we aim to investigate the differences in and relationship between CMJ and FK performances with and without a carried load in military personnel. Given the previously observed importance of maximal and explosive lower limb strength in performing the front kick, we predict that there will be positive correlations between 1RM BS, CMJ, and FK. We further hypothesize (43) that the carried load will not only negatively affect CMJ and FK performances but also weaken their relationship compared with the unloaded conditions.

## Methods

### Experimental Approach to the Problem

**Figure 1.**
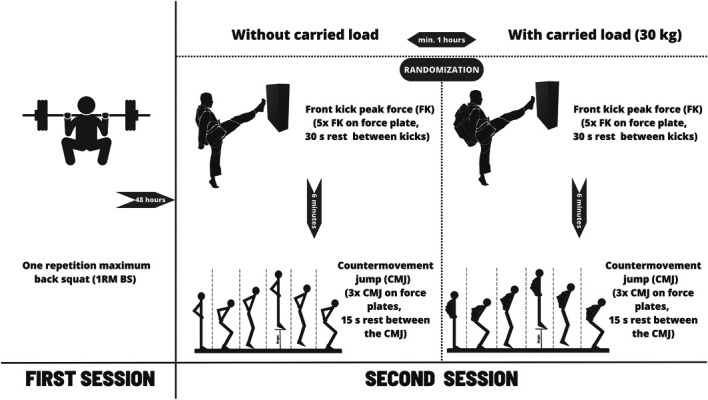
Timeline of the study protocol.

This study specifically investigates the relationship between FK performance and the maximal strength (as measured by the 1RM BS) and explosive strength (as measured by CMJ) of the lower limbs with and without carried load. Participants took part in 2 data collection sessions 48 hours apart (Figure 1). In the first session, they reported their age, body height, and body mass, followed by a standardized and supervised warm-up. Subsequently, under the supervision of a certificated Strength and Conditioning (NSCA-CSCS) coach, they began with the back squat protocol to reach a personal 1RM BS. During the second session (after a standardized and supervised warm-up), the FK and CMJ performances were measured with and without a carried load. Thus, each participant repeated the same protocol twice. The order of performance (FK or CMJ) was kept constant, but the test conditions (with × without carried load), with at least an hour break between them, were randomized between participants.

### Subjects

The study was conducted during Q2 of 2022 in collaboration with the Biomechanics of Extreme Loads and Training Adaptation laboratories at the Faculty of Physical Education and Sport. All procedures were conducted following the Declaration of Helsinki, and the Institutional Review Board of the Faculty of Sport and Physical Education approved the study (085/2022). All participants were informed about the study's purpose and signed an informed consent form expressing their willingness to take part voluntarily.

As the main aim of this investigation is directed toward military personnel and their training, participants were recruited among the Department of Military Physical Education cadets at the Faculty of Physical Education and Sport. At the time, the department had a total of 23 cadets. From these, only those who met the following criteria were eligible to participate: being 18 years of age or older, meeting medical classification “A” (able to perform military service without limitation), not suffering an acute illness, having no musculoskeletal injuries in the last 3 months, having experiences with the selected tasks (back squat, front kick), and carrying gear loads. All cadets in the department underwent a hand-to-hand combat course covering front kicks and mastering the technique comparable with subelite athletes. They were also introduced to proper squatting technique in the troop's introductory physical training lessons, which were led by an NSCA CSCS coach. Carrying ballistic armor and a loaded military backpack is standard practice across multiple introductory and military skills–oriented courses in the department's curriculum.

A total of 22 participants met our criteria. However, one participant was excluded from the study because of missing data from the force plate and, therefore, could not be evaluated. The final study sample consists of 21 participants; for detailed sample descriptions see Table 1.

**Table 1:** Descriptive statistics of the sample.*

	Mean	*SD*	Minimum	Maximum
Body mass (kg)	81.33	9.56	60	96
Height (cm)	181.38	6.23	170	194
Age (y)	22.76	1.97	20	27
1RM BS (kg)	129.12	20.63	90	160
FK without carried load (N)	6,259.50	1,493.80	3,237.14	9,240.96
FK with carried load (N)	5,083.44	1,135.23	2,580.51	7,762.04
CMJ without carried load (cm)	37.60	4.91	28.38	48.47
CMJ with carried load (cm)	23.40	4.367	11.06	38.72

* 1RM BS = one repetition maximum back squat; FK = front kick peak force; CMJ = countermovement jump.

### Procedures

We used within-participants experimental design with repeated measures. This approach involved conducting a series of performance tests—1RM BS, CMJ, and FK, both with and without carried load.

#### One Repetition Maximum Back Squats

To estimate each participant's 1RM BS, we followed the protocol and technique used in previous studies (35,48). Each participant performed a standardized and supervised warm-up (5 minutes of riding on the exercise bike, followed by a 10-minute dynamic warm-up focusing on the lower limbs) to prepare for the task and reduce potential risks of injury. Subsequently, they began with supervised 1RM BS. The protocol (35) consisted of 2 sets of 5 repetitions at 40–60% of self-reported 1RM BS (2-minute rest between sets), followed by 3-minute rest and 2 sets of 2–3 repetitions at 60–80% of 1RM BS (2-minute rest between sets). Consecutive single maximal attempts with increasing load were then performed (3–5 minutes rest between attempts) until the participants reached their 1RM BS. Protocol was terminated in the event of 2 failed attempts or when the participant could not maintain the correct movement pattern of the back squat. The successful maximum performance in kilograms (1RM BS) (Table 1) was used for subsequent analysis.

The squats were carried out in a weightlifting squat rack using a calibrated Eleiko bar (20 kg, 28 mm bar diameter) with calibrated Eleiko weight plates and collars. Only the bar weight and weight plates were summed for the total weight; the collars' weight was neglected.

#### Front Kick Peak Force

Participants started with a standardized supervised warm-up consisting of dynamic stretching, practice kicks, jumps, squats, toe touches, and CMJ exercises, lasting 10 minutes (3,62). After the warm-up, the participants wore standard-issue Czech Army military boots (Prabos 2000) (63). They wore tight athletic leggings and t-shirts, allowing for a free and full range of motion. They were instructed to initiate the front kick from a fighting stance and always from the back foot (52). They were to raise the knee of their dominant leg directly forward while bringing the foot toward the hip joint and then extend their leg forcefully to deliver the strike with the sole of the boot (40), targeting mid-height (typically the abdomen or solar plexus region). They performed 5 practice front kicks, gradually increasing intensity to familiarize themselves with the force plate. Each participant kicked from a self-selected distance from the force plate, allowing for a natural and preferred kicking position. These individualized distances were noted and used to ensure consistent starting positions for each consecutive trial. Participants performed one set of 5 front kicks with and without a carried load (Figure 1). Each kick was followed by a 30-second rest period, and after the completion of the fifth kick, participants had a 6-minute rest (62,63).

The measurement was carried on a three-axial force plate model Kistler 9286BA (Kistler, Winterthur, Switzerland) consisting of 600 × 400 × 35 mm aluminum sandwich top plates with 4 built-in piezoelectric 3-component force sensors. The force plate was connected to a computer with a 16-bit A/D board and BioWare V5.3.2.9 software. The force plate recorded the dynamics of each front kick at a sampling rate of 1,000 Hz (51). The signals from the force measurement pads were amplified and stored for further analysis. A setting of 100 N was applied to minimize noise in the data and to indicate the initiation and conclusion of the interaction between the participant's foot and the force plate (23). A tatami mat (400 × 300 × 25 mm, StrongGear) was attached to the force plate face to reduce the risk of injury. The force plate was mounted vertically on a wall 1.25 meters above the ground, providing a plate height corresponding to the participants' average height (37). During the experiment, the peak forces (in N) generated by each front kick (Table 1) were extracted from the recorded data using MATLAB software (version 1.8.0.121; MathWorks, Natick, MA) (17,62).

#### Countermovement Jump

After a 1-hour rest, participants performed the same warm-up routine as before the FK protocol (3,62). After the warm-up, the CMJ familiarization session commenced, during which information on how to perform the CMJ was provided, followed by 3 practice attempts. They were instructed to initiate the countermovement, immediately attempt to jump vertically as high as possible, and return to standing upon impact. Participants were to keep their hands on their hips throughout the jump to focus more on the force generated by the lower limb by limiting their arm movements (26).

After that, the CMJ protocol started, and participants performed 3 CMJs and rested for 15-second between trials (8). Each trial began with participants standing still with both feet on one force plate. Each trial was monitored for erroneous attempts, and a trial was discarded if the participant could not land each foot on the force plate or returned to standing upon impact. As per Barker et al. (3), 6 trials were provided to complete 3 successful trials. All participants managed to complete the 3 CMJ trials. CMJ was measured using 2 three-axis force plates Kistler 9286BA (Kistler, Winterthur, Switzerland) placed side by side at a 1,000 Hz sampling rate (51). They were connected to a computer using a 16-bit A/D board and BioWare V5.3.2.9 software to amplify and record the signals from both plates. Each trial's jump height (cm) was obtained from flight time and extracted using MATLAB (1.8.0.121; MathWorks, Natick, MA) (17).

#### Carried Load

For the carried loaded conditions, each participant wore standard-issued military ballistic body armor and a military backpack. The ballistic body armor (total mass 10 kg) consisted of a plate carrier, including front and back plates (weighing 6.5 kg) and a pouch (3.5 kg) placed on the front of the vest, mimicking an ammunition magazine pouch. The military backpack, weighing 20 kg, contributed to a total carried load of 30 kg. This load was explicitly chosen to represent a typical load (4) for military personnel, which can often constitute up to 60% of their body mass, and not to interfere with the front kick technique (i.e., participants can maintain the front kick movement pattern with this carried load (58)).

### Statistical Analyses

Statistical analyses were performed in JASP (0.16.2) software (28). Using Q-Q plots and the Shapiro-Wilk test, we verified the assumptions of normality in our data (1RM BS, FK, and CMJ, both with and without carried load). To assess performance consistency across repetitions in FK and CMJ with and without carried load, we calculated type ICC (1,2) intraclass correlations (45), reported with their 95% CI [LL, UL], and also coefficient of variation (CV) (18). We interpret ICC values <0.5 as poor, 0.5–0.75 as moderate, 0.75–0.9 as good, and >0.9 as excellent consistency (22). Similarly, we interpret CV values ≤10% as very good, indicating low variability in measurements; 10%–20% as acceptable; and >20% as high (18). Overall repeatability was classified as very high (CV ≤ 5%, ICC ≥ 0.95), high (CV ≤ 10%, ICC ≥ 0.90), and moderate (CV ≤ 15%, ICC ≥ 0.80), by previous reliability studies (6). We deemed values of CV ≤ 15% and ICC ≥ 0.80 as a threshold of sufficient consistency. For all further analyses, all 5 front kicks with and without carried load and 3 CMJ heights (cm) with and without carried load were aggregated into mean FK with and without load and mean CMJ with and without load.

Next, we used Pearson's *r* (one-tailed) and its 95% CIs to explore the relationship among the FK with and without carried load, CMJ with and without carried load, and 1RM BS. We interpret the strengths of the associations as trivial |0–0.1|, small |0.1–0.3|, moderate |0.3–0.5|, large |0.5–0.7|, or very large |0.7–0.9| (19). Using paired samples *t* tests (one-tailed), we analyzed differences in mean performances with and without carried load conditions for FK and CMJ. The mean differences and their Cohen's *d* equivalent with 95% CIs are reported.

Finally, we ran a linear regression model to test how 1RM BS and CMJ performances are associated with FK. We fitted a second model to test how CMJ with carried load is associated with FK with carried load. Model fits are reported as R^2^, and both unstandardized and standardized beta slope estimates with 95% CIs are reported for all associations.

#### Sensitivity Analyses

Because of the restricted available sample population (25), we calculated a sensitivity analysis for all planned tests with *N* = 21 in G*Power (v 3.1.9.6). In the case of (Exact) bivariate correlations, with 5% α and 20% β levels (i.e., 80% power) and one-tailed test, we have sensitivity to observe correlations with *r* ≥ 0.51. For paired-sample t-tests, with 5% α and 20% β levels (i.e., 80% power) and 2-tailed test, we have sensitivity to observe differences in the magnitude of Cohen's *d* ≥ 0.562. In a fixed linear regression model with 2 associated variables, 5% α and 20% β levels (i.e., 80% power), we have sensitivity to observe *R*^2^ ≥ 0.544 and *R*^2^ ≥ 0.415 in the model with one associated variable.

#### Data availability and supplemental digital content

Project registration, associated dataset, and analysis results are publicly available on the Open Science Framework (OSF) repository (see Data Availability, Supplemental Digital Content 1, https://osf.io/jef64).

#### Artificial Intelligence

Throughout preparing an early draft, the primary author used Open AI ChatGPT-4 and Grammarly to improve spelling, grammar, sentence structure, and paragraph flow. After using these tools, all authors proofread, reviewed, and edited the content as needed. Open Ai's ChatGPT-4 was also used to plot and enhance figures (e.g., sharpening visuals, improving colors, clarity, and readability) based on the results of the JASP analysis (e.g., mean and CI values).

## Results

### Performance Consistency

**Figure 2.**
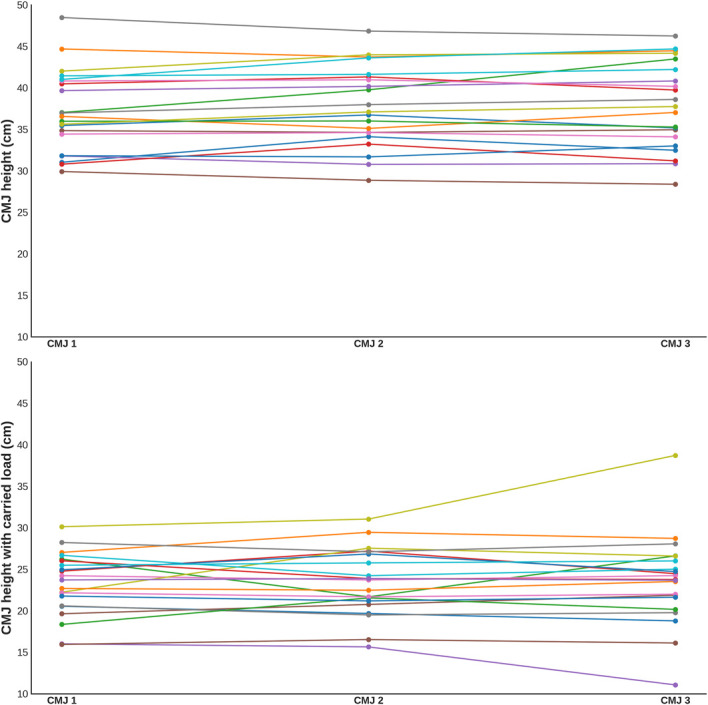
The performance across CMJ trials, without and with carried load. Color dots and lines represent individual subjects and their attempts. CMJ = countermovement jump.

The performances across FK trials, both without and with carried load, showed moderate levels of consistency with ICC (2,1) FK No load = 0.666 (0.489–0.822) and ICC (2,1) FK Carried load = 0.555 (0.364–0.747), respectively. These FK trials also exhibited high variability, with CV FK No load values of 20.7% and CV _FK Carried load_ of 18.1%, indicating a lower reliability and significant differences between individual measurements. In contrast, the CMJ trials showed excellent consistency without carried load (ICC (2,1) CMJ No load = 0.947 [0.895–0.976]) and good consistency with carried load (ICC (2,1) CMJ Carried load = 0.855 [0.730–0.933]). The variability in CMJ trials was more acceptable, with CV _CMJ No load_ values of 13% without carried load and CV _CMJ Carried load_ of 18.6% with carried load, reflecting sufficient reliability for practical purposes (Figure 2).

### Associations Between Outcomes of Countermovement Jumps, One Repetition Maximum Back Squat, and Front Kicks

Countermovement jumps and FK performances with and without carried load were strongly positively and statistically significantly associated (Table 2). With a similarly strong association, CMJ without carried load was statistically significantly associated with 1RM BS. However, although positive, the FK without carried load was not associated with 1RM BS statistically significantly (Table 2). Although statistically significant, our sensitivity to observe these associations between CMJ without carried load and FK without carried load or 1RM BS was below 80%. An alternative approach could be to look at relative 1RM BS strength normalized to individual body mass. These results can be found in the Supplemental Digital Content 1 (see Data Availability, https://osf.io/jef64). We found that the pattern of results remains consistent regardless of whether absolute or relative strength measures are used, indicating that either method yields comparable insights.

**Table 2:** Associations among one repetition maximum back squat, countermovement jump, and front kick peak force (without and with carried load).*

Variables	Pearson's *r* (*n* = 21)	*p*	95% CI
CMJ without—CMJ with carried load	0.624	0.001	0.330 to 1.000
FK without—FK with carried load	0.736	<0.001	0.503 to 1.000
1RM BS—CMJ without carried load	0.544	0.005	0.219 to 1.000
1RM BS—FK without carried load	0.199	0.193	−0.184 to 1.000
CMJ without carried load—FK without carried load	0.549	0.005	0.225 to 1.000
CMJ with carried load—FK with carried load	0.266	0.122	−0.115 to 1.000

* CMJ = countermovement jump; FK = front kick peak force; 1RM BS = one repetition maximum back squat.

### Differences in Performance Without and With Carried Load

**Figure 3.**
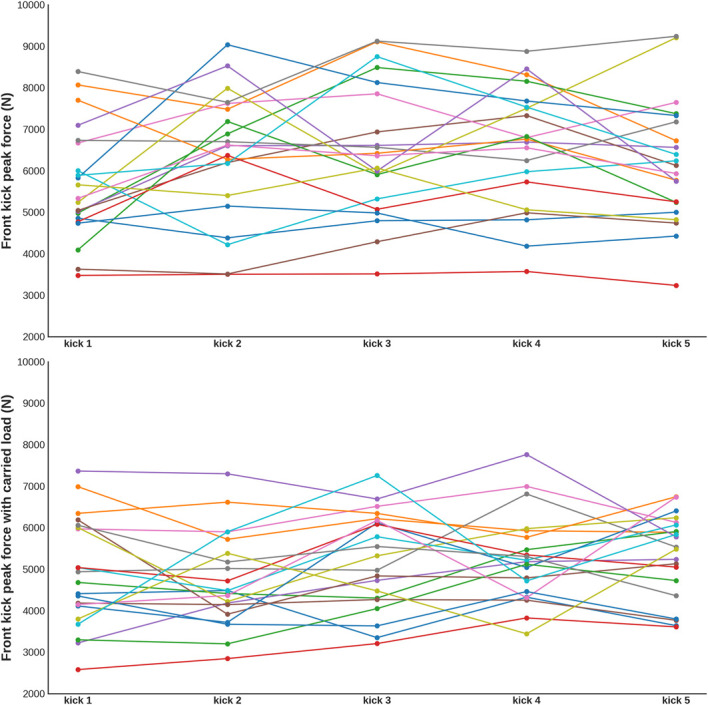
The performance across FK trials, without and with carried load. Color dots and lines represent individual subjects and their attempts. FK = front kick peak force.

We observed an expected statistically significant decrease in mean performance with carried load compared to without carried load in the CMJ (Cohen's *d* = 3.55 [2.543–∞], *t_20_* = 16.266 [12.692-∞] *p* < 0.001) (Figure 3, left panel). Similarly, FK was also statistically significantly lower compared to without carried load (Cohen's *d* = 1.339 [0.828–∞], *t_20_* = 6.134 [843.15-∞] *p* < 0.001) (Figure 3, right panel).

### Linear Regression Model

**Figure 4.**
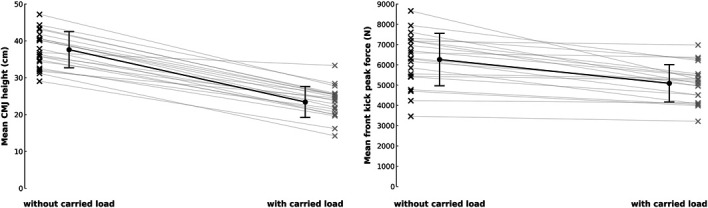
Mean differences in performance without and with carried load. Full black circles indicate means and error bars their 95% confidence interval. “X” represents individual performances; gray lines connect the performance points of the same participant across conditions. Black X denotes “without carried load” and gray × “with carried load” conditions.

The linear regression model with 1RM BS and CMJ without carried load as associated variables was statistically significant (F_2, 18_ = 4.142, *p* = 0.033) and accounted for 31.5% of the observed variance (*R*^2^ = 0.315) of the mean FK without carried load. Out of the 2 associated variables, only CMJ without carried load was statistically significant (*p* = 0.015) and had a positive slope (Table 3, Figure 4).

**Table 3:** Linear regression of one repetition maximum back squat and countermovement jump without and with carried load associating with front kick peak force.*

Model	Predictors	Unstandardized estimate	*SE*	Standardized estimate	T	*p*	95% CI
Without carried load	Intercept	1,195.671	2049.707		0.583	0.567	−3,110.603 to 5,501.945
	1RM BS	−8.868	14.582	−0.141	−0.608	0.551	−39.504 to 21.768
	CMJ	165.141	61.375	0.626	2.691	0.015	36.198 to 294.085
With carried load	Intercept	3,722.933	1,152.166		3.231	0.004	1,211.421 to 6,134.445
	CMJ	58.267	48.491	0.266	1.202	0.244	−43.225 to 159.759

* 1RM BS = one repetition maximum back squat; CMJ = countermovement jump.

**Figure 5.**
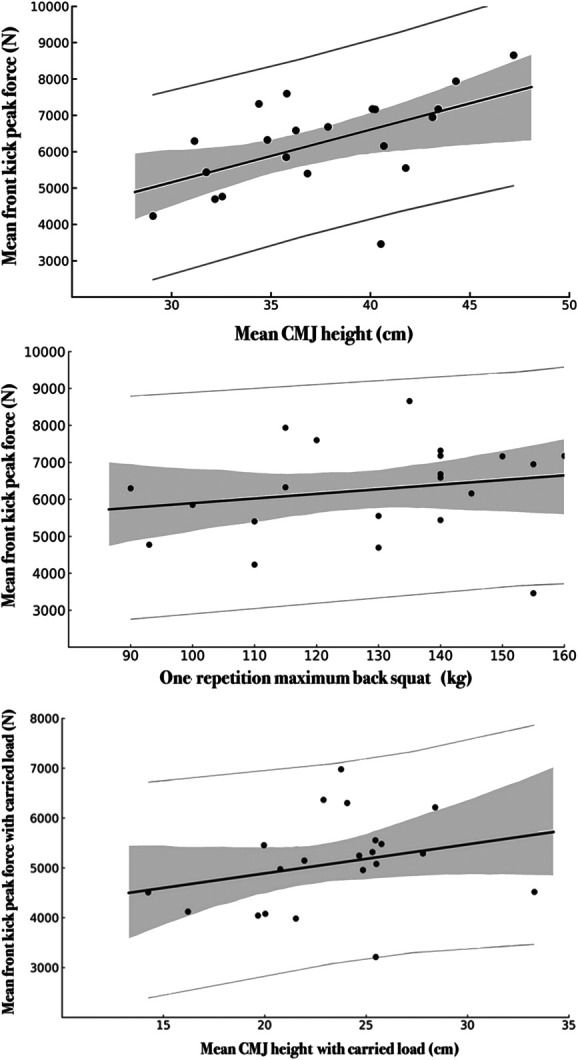
Linear regression of one repetition maximum back squat and countermovement jump without (top and middle) and with carried load (bottom) associating with front kick peak force. The full black line represents the estimated slope, and the gray bands represent 95% CI. The gray lines depict the prediction interval. Black dots indicate individual observations.

The linear regression model with only CMJ with carried load as the associated variable was not statistically significant (F_1, 19_ = 1.444, *p* = 0.244) and accounted only for 7.1% of the variance (*R*^2^ = 0.071) of the mean FK with carried load (Table 3, Figure 5).

## Discussion

Our research findings show that explosive strength (measured by CMJ without a carried load) and maximal strength (measured by 1RM BS) of the lower body explained 31.5% of the variability in FK when performed without a carried load. However, only lower limb explosive power without carried load was statistically significantly associated with the FK. Neither the 1RM BS nor the CMJ with the carried load serves as a reliable indicator for the FK, regardless of whether the FK is executed with or without a carried load.

Our initial hypothesis was that there would be discernible positive correlations between 1RM BS, CMJ, and FK, and that the introduction of a carried load would adversely affect both CMJ and FK performances, thereby weakening their interrelationship in comparison with conditions without a load. The outcomes of our study, however, demonstrate that the 1RM BS was not associated with FK performance, and the association between CMJ and FK fades when a carried load is introduced. Our results corroborate the prediction that carrying a load has a negative impact on both CMJ and FK performance, underscoring the complex interplay between physical strength, explosive power, and technical skill in executing military tasks under various conditions.

Previous studies have demonstrated moderate to strong correlations between 1RM BS and CMJ (r_25_ = 0.87 (36), r_26_ = 0.69 (30), r_16_ = 0.52 (64)), we observed a similar correlation between 1RM BS and CMJ in our study (*r* = 0.54 [0.219–1]). In addition, we found a moderate positive correlation between CMJ and FK (*r* = 0.55 [0.225–1]). However, our results indicate that 1RM BS is not significantly associated with FK performance, and the correlation between CMJ and FK diminishes when a load is introduced. These findings support the prediction that carrying a load negatively impacts both CMJ and FK performance, highlighting the complex interplay between physical strength, explosive power, and technical skill in executing military tasks under varying conditions.

Although we found a positive correlation between 1RM BS and CMJ, this correlation was not observed between 1RM BS and FK without the carried load. This discrepancy may stem from biomechanical differences inherent to these exercises: the squat involves a loaded, controlled movement emphasizing strength, whereas the front kick is an unloaded, rapid movement prioritizing speed and technique. The differential demands of these movements on muscle recruitment, neuromuscular coordination, and energy utilization likely influenced the outcomes. Although strength certainly plays a role, it did not manifest in this case. When kicking an opponent, we aim not to push them away with maximum (slow) strength but to kick them away dynamically. Although these results may come as surprising, they make sense when considering the dynamic nature of kicking versus the more static nature of squatting.

In our sample, the ICCs showed excellent and good consistency for CMJ for CMJ without and with carried load, respectively. These results are in line with findings from other research (24,42). Previous studies reported good to excellent levels of consistency in FK. However, in the case of FK, we observed ICCs of only moderate consistency in both with and without carried load conditions, contrasting to previous studies showing good to excellent levels of ICCs (59). Lower FK consistency in our sample may stem from differences in technique maturity of our participants compared with other studies. Participants of previous studies reporting higher ICCs were frequently an elite group that can perform FK more consistently than our sample of a subelites (44).

It is worth noting that comparing our ICC results with other studies is difficult as many either use different or do not specify the ICC they report (59). In our study, ICC(2,1) was chosen to assess the consistency of individual kicks across multiple trials by the same participant, offering a more detailed understanding of variability and reliability. This method is crucial for evaluating true performance consistency. Although the ICC for FK with carried load was only moderate (ICC = 0.555 [0.364–0.747]), the CV indicated an acceptable level of variability (CV = 18.1%). In contrast, the ICC for FK without load was higher (ICC = 0.666 [0.489–0.822]), yet the CV was also higher (CV = 20.7%). These findings suggest that although the consistency without load was better, the relative variability (CV) was greater, indicating somewhat greater performance dispersion in performance without load. These findings suggest that the CV values were consistent with results from other studies, indicating an acceptable level of variability. For CMJ, we found that with and without a carried load, the CVs were (18 and 13%, respectively) indicating acceptable variability and high consistency, consistent with other research (42).

Previous studies show moderate to strong positive correlations between 1RM BS and CMJ (*r*_25_ = 0.87 (36), *r*_26_ = 0.69 (30), *r*_16_ = 0.52 (64)). We also observed a comparable correlation between the 1RM BS and CMJ (*r* = 0.54 [0.219–1]) in our study. There was also a moderate positive statistically significant correlation between CMJ and FK (*r* = 0.55 [0.225–1]). Although maximal strength is considered a crucial performance measure in combat sports such as MMA (21), 1RM BS and FK were not statistically significantly associated in our sample.

Countermovement jumps are a widely recognized reliable diagnostic tool for assessing lower body explosive strength, a crucial component in achieving maximum power and speed across sports (46). Countermovement jumps performance also positively correlates with occupational performance, particularly among military personnel during training and tactical operations (31,33). The dynamic motion of the lower limbs during CMJ is likely closely related to the kinetic patterns observed in techniques such as the front kick (38). This relationship is further highlighted in roundhouse kicks (14), where a significant positive correlation (*r*_31_ = 0.771) was found between CMJ performance and the kick velocity. Hip motion seems to be a primary driver of dynamic forces in both CMJ and various kicking techniques (58), including the front kick, highlighting the role of hip motion in the development of explosive strength necessary for better performance in both CMJ and kicking actions.

In the present study, participants underwent additional sets of CMJ and FK while carrying a 30-kg weight simulating military operational loads. As expected, the carried load resulted in a substantial performance decrement—37.8 and 18.8% decrease for the CMJ and FK, respectively. These results are in line with previous evidence observing drops in CMJ by 26.5 and 41% with carried loads of 25 and 50% body mass, respectively, compared with no load (24). The observed performance decrements could be attributable to alterations in body movements caused by the carried load and its distribution (e.g., carrying a loaded backpack) affecting individual's center of mass, potentially impeding balance, efficiency, and consistency to generate high peak forces in movements like front kick (9,39,54). Performance and consistency in movements like the front kick depend on the individual's ability to control shifts in the body's center of mass. This skill gained through practice and training may not fully translate under novel or altered conditions, like carrying an additional load. As our participants had no previous experience performing CMJ and FK with a carried load, their performance and consistency were thus affected and subpar.

Similarly, a study by Merrigan et al. (32) observed changes in CMJ performers with added load reducing the jump height. Interestingly, we no longer observed the statistically significant positive correlation between CMJ and FK in the carried load condition. This may be because FK is a more demanding technique (supported by the lower performance consistency measures) than CMJ. The carried load likely exacerbates this issue, resulting in even lower reliability for FK in the carried load condition. Consequently, the carried load may not have allowed participants to perform at the level of their true maximal peak force abilities, which could have dampened the observed association.

The strength of our study and the effects observed lie in the homogeneity of our sample. Nonetheless, we point out the final sample was relatively small, although we included all eligible members from our department as participants. The sample size ultimately limits our sensitivity to reliably detect the observed magnitudes of results. Although our small sample size may limit statistical significance, it is important to focus on the strength of the associations found and their practical value (e.g., rather low R^2^ values). Thus, our findings and their practical implications should be viewed as somewhat limited and asking for further extensions.

Our goal was to include participants who had sufficiently mastered the front kick technique and had experience with strength training. Although they are competent in the FK technique for military applications, they may not have mastered it to a level comparable with elite or subelite sports groups. This is primarily caused by training oriented toward military objectives rather than the competitive sports framework.

To address the observed inconsistencies in performance when subjects are equipped with operational gear, future research should focus on how targeted maximal and explosive strength training, specifically adapted for execution in military gear, impacts FK. Therefore, it is worth considering the recommendation that training under loaded conditions, simulating real field experiences, be incorporated to enhance the relevance of training protocols and reduce performance variations, thereby better preparing military personnel for the physical demands of real-world scenarios.

**Practical Applications**

From a practical standpoint, the study suggests that explosive power (measured by CMJ) shows a better association with the peak force of the front kick than the maximal strength (measured by 1RM BS). Thus, training programs focused on lower limb explosive power could allow athletes and military personnel to achieve higher FK. Combat sports trainers and hand-to-hand combat instructors may thus consider emphasizing plyometrics and other explosive training methods for enhancing the peak force of kicks.
